# Failure of thymic deletion and instability of autoreactive Tregs drive autoimmunity in immune-privileged liver

**DOI:** 10.1172/jci.insight.141462

**Published:** 2021-03-22

**Authors:** Max Preti, Lena Schlott, David Lübbering, Daria Krzikalla, Anna-Lena Müller, Fenja A. Schuran, Tobias Poch, Miriam Schakat, Sören Weidemann, Ansgar W. Lohse, Christina Weiler-Normann, Marcial Sebode, Dorothee Schwinge, Christoph Schramm, Antonella Carambia, Johannes Herkel

**Affiliations:** 1 Department of Medicine I,; 2 Department of Pathology, and; 3 Martin Zeitz Center for Rare Diseases, University Medical Centre Hamburg-Eppendorf, Hamburg, Germany.

**Keywords:** Autoimmunity, Hepatology, Autoimmune diseases, Hepatitis, T cells

## Abstract

The liver is an immune-privileged organ that can deactivate autoreactive T cells. Yet in autoimmune hepatitis (AIH), autoreactive T cells can defy hepatic control and attack the liver. To elucidate how tolerance to self-antigens is lost during AIH pathogenesis, we generated a spontaneous mouse model of AIH, based on recognition of an MHC class II–restricted model peptide in hepatocytes by autoreactive CD4^+^ T cells. We found that the hepatic peptide was not expressed in the thymus, leading to deficient thymic deletion and resulting in peripheral abundance of autoreactive CD4^+^ T cells. In the liver, autoreactive CD4^+^ effector T cells accumulated within portal ectopic lymphoid structures and maturated toward pathogenic IFN-γ and TNF coproducing cells. Expansion and pathogenic maturation of autoreactive effector T cells was enabled by a selective increase of plasticity and instability of autoantigen-specific Tregs but not of nonspecific Tregs. Indeed, antigen-specific Tregs were reduced in frequency and manifested increased IL-17 production, reduced epigenetic demethylation, and reduced expression of *Foxp3*. As a consequence, autoantigen-specific Tregs had a reduced suppressive capacity, as compared with that of nonspecific Tregs. In conclusion, loss of tolerance and the pathogenesis of AIH were enabled by combined failure of thymic deletion and peripheral regulation.

## Introduction

Autoimmune diseases are caused by a tissue-damaging immune response of lymphocytes recognizing self-antigens (1). The clinical conditions caused by autoimmunity often impose severe health burdens on affected individuals and collectively represent a major economic burden for healthcare systems (1). Although many autoreactive T cells are efficiently deleted at an immature state in the thymus, it has been demonstrated that self-reactive T cells are a constitutive part of the mature lymphocyte repertoire in healthy individuals (2–4). Nonetheless, the activation of these mature autoreactive T cells seems to be tightly controlled by various mechanisms, first and foremost by Tregs (4–6). Hence, autoreactive T cell activation rarely produces more than subclinical, self-limited autoimmune episodes in most subjects. The reasons why some individuals develop self-sustained autoimmune diseases, despite these regulatory mechanisms, are not clear.

A particular challenge is to conceptualize those autoimmune diseases that affect immune-privileged organs, such as the liver, which actually has a distinct capacity to induce immune tolerance (7, 8). Indeed, the liver can even protect other organs from immune-mediated damage, as has been demonstrated by cotransplantation of skin and liver allografts (9) or by complete protection from autoimmune neuroinflammation induced by genetic or nanoparticle-mediated transfer of neuroantigens to liver cells (10, 11). Given this potent tolerance-inducing capacity of the liver, it is surprising that the liver cannot protect itself from autoimmune attack in autoimmune liver diseases, such as autoimmune hepatitis (AIH).

AIH is a chronic inflammatory liver disease that can occur in people of all ages and ethnicities, with heterogeneous clinical manifestations ranging from inconspicuous presentation up to fulminant liver failure (12). Typical disease features include elevated serum transaminases; elevated IgG or γ globulins; the presence of autoantibodies, such as antinuclear antibodies (ANAs); and a histological picture of interface hepatitis with mainly lymphocytic infiltrates (12). Thus far, the etiology and pathogenesis of AIH are unclear, but it is assumed that AIH is driven by an adaptive immune response to hepatocellular autoantigens (12). Indeed, several antigen-driven AIH mouse models have demonstrated that T cell responses to liver antigens can cause liver inflammation (13–19). Nonetheless, the mechanisms that produce the loss of tolerance to liver antigens in AIH are not fully understood. A drawback of the available mouse models is the difficulty to characterize and distinguish autoantigen-specific from nonspecific T cells. Moreover, the majority of the available AIH mouse models requires extrinsic provision of strong immune activators to overcome hepatic tolerance (13, 19), which might not reflect the natural disease development.

It is now recognized that the potent immune activation in autoimmune diseases often occurs within ectopic (or tertiary) lymphoid tissue (ELT) that develops in the target organs of autoimmunity (20). ELTs are ordered structures induced within affected organs that resemble the organization of lymphoid follicles in secondary lymphatic organs, frequently displaying segregated T cell and B cell zones, presence of DCs, and high endothelial venules (21). In several chronic inflammatory liver diseases, ELTs can be found mostly within portal fields (22, 23). However, little is known about a possible role of ELTs in AIH pathogenesis.

Besides being driven by strong activation of effector T cells, AIH might also be associated with reduced functionality of Tregs. Tregs are critical mediators of self-tolerance, and their loss or dysfunction can lead to autoimmune disease (6). Several observations (reviewed in refs. 24, 25) suggest that Tregs have the potential to modulate AIH. However, their precise role in AIH pathogenesis is unclear, as contradictory findings with respect to their overall frequency and functionality have been reported (26, 27). Intriguingly, hepatic infiltrates in AIH reportedly contain Tregs (27–29), suggesting that the inflammatory environment in AIH livers may override the suppressive capacity of the hepatic Tregs (30, 31). Indeed, Tregs can display considerable plasticity that allows for adaptation to various physiological conditions (32, 33); however, in the context of autoimmune liver inflammation, this plasticity might result in impaired function.

To further explore the mechanisms underlying the loss of tolerance to liver antigens in AIH, we here investigate a new mouse model that is characterized by hepatocellular expression of an MHC class II–restricted immunodominant T cell epitope of the lymphocytic choriomeningitis virus (GP61–80), and by abundance of cognate CD4 T cells recognizing GP61–80 expressed by hepatocytes. These mice spontaneously developed CD4^+^ T cell–mediated AIH with typical disease features of human AIH. We demonstrate that AIH is driven by escape from thymic deletion and hepatic activation of CD4^+^ effector T cells, which was fostered by selective failure of peripherally induced autoreactive Tregs.

## Results

### Absent thymic deletion of CD4^+^ T cells recognizing a liver-restricted peptide.

**Figure 1:**
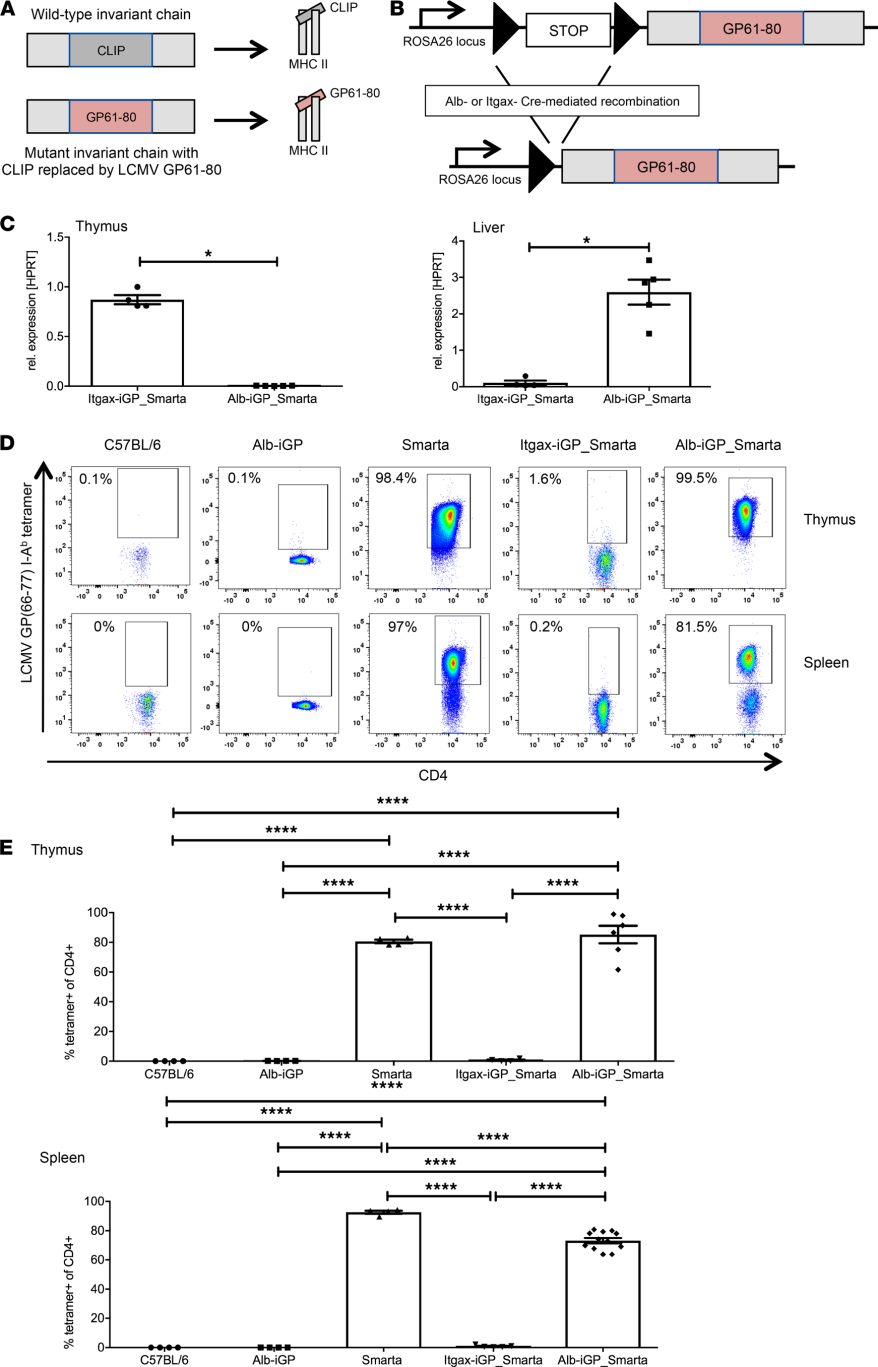
Generation of Alb-iGP_Smarta mice and characterization of thymic and peripheral autoreactive CD4^+^ T cells. (**A**) Scheme of the Alb-iGP system. The CLIP sequence in the CD74 gene was replaced by the GP61–80 sequence, facilitating high occupancy of MHC II molecules and GP61–80 presentation. (**B**) Cre-mediated removal of a STOP cassette facilitates expression of the mutant CD74 molecule under Rosa26 promoter control. (**C**) GP61–80 expression levels in thymus and liver of Itgax-iGP_Smarta and Alb-iGP_Smarta mice, as determined by quantitative RT-PCR relative to the HPRT housekeeper gene expression. (**D**) Representative flow cytometry dot plots of antigen-specific I-A(b) GP66–77 tetramer–binding CD4^+^ T cells in thymus (top) or spleen (bottom) in C57BL/6 mice, Alb-iGP mice expressing the mutant CD74 molecule, Smarta mice expressing a transgenic T cell receptor recognizing the cognate GP61–80 peptide, Itgax-iGP_Smarta mice, or Alb-iGP_Smarta mice, featuring both presentation of the GP61–80 peptide and the cognate Smarta T cell receptor. (**E**) Thymic (top) and splenic (bottom) frequencies of I-A(b) GP66–77 tetramer–specific CD4^+^ T cells in C57BL/6, Alb-iGP, Smarta, Itgax-iGP_Smarta, or Alb-iGP_Smarta mice. Data are shown as the mean ± SEM (*n* = 4–12). *P < 0.05; ****P < 0.0001 (**C**, Mann-Whitney; **E**, ANOVA).

We constructed a mutated invariant chain molecule (CD74) in which the CLIP peptide sequence was replaced by the LCMV GP61–80 peptide sequence and inserted the mutated gene flanked by loxP sites into the *Rosa26* gene, as has been done before with a similar construct (34) (Figure 1A). These mice with inducible glycoprotein (GP) peptide (iGP) expression were bred with Smarta mice that are transgenic for the Smarta1 T cell receptor recognizing the GP61–80 peptide (35). Conditional expression in hepatocytes was subsequently achieved by breeding with Alb-Cre mice; as control, conditional expression in DCs was achieved by breeding with Itgax-Cre mice (Figure 1B). The resulting Alb-iGP_Smarta and Itgax-iGP_Smarta mice were analyzed by quantitative RT-PCR for expression of the recombinant GP peptide in the thymus and in the liver (Figure 1C). As expected, GP expression was low in Itgax-iGP_Smarta livers and high in Alb-iGP_Smarta livers (0.10 vs. 2.59; *P* = 0.0159). In contrast, GP expression was high in Itgax-iGP_Smarta thymi and virtually absent in Alb-iGP_Smarta thymi (0.87 vs. 0.039; *P* = 0.0159). We then analyzed Itgax-iGP_Smarta and Alb-iGP_Smarta mice for deletional tolerance mechanisms, using I-A(b) LCMV GP 66–77 tetramers that specifically stain those T cells recognizing the GP peptide (Figure 1D). We found that virtually all GP-reactive T cells were subjected to thymic deletion in Itgax-iGP_Smarta mice (Figure 1E; 0.68%; *P* < 0.0001). Accordingly, GP-reactive CD4^+^ T cells were negligible in the spleen of Itgax-iGP_Smarta mice (0.85%; *P* < 0.0001). In contrast, there was almost no deletion of GP-reactive Smarta cells in the thymi of Alb-iGP_Smarta mice, which had a similar frequency of antigen-specific cells as Smarta control mice that do not express the cognate GP peptide (Figure 1E; 85.3% vs. 80.6%; *P* = 0.8414). Accordingly, only a moderate reduction of GP-reactive CD4^+^ T cells was found in the spleens of Alb-iGP_Smarta mice (73.2% vs. 92.7% in Smarta mice; *P* < 0.0001). Thus, the GP61–80 peptide did not seem to trigger extensive deletion of the specific CD4 Smarta cells in Alb-iGP_Smarta mice, neither in the thymus nor in the periphery.

### Spontaneous AIH in mice featuring autoantigen peptide in the liver and peripheral autoreactive T cells.

**Figure 2:**
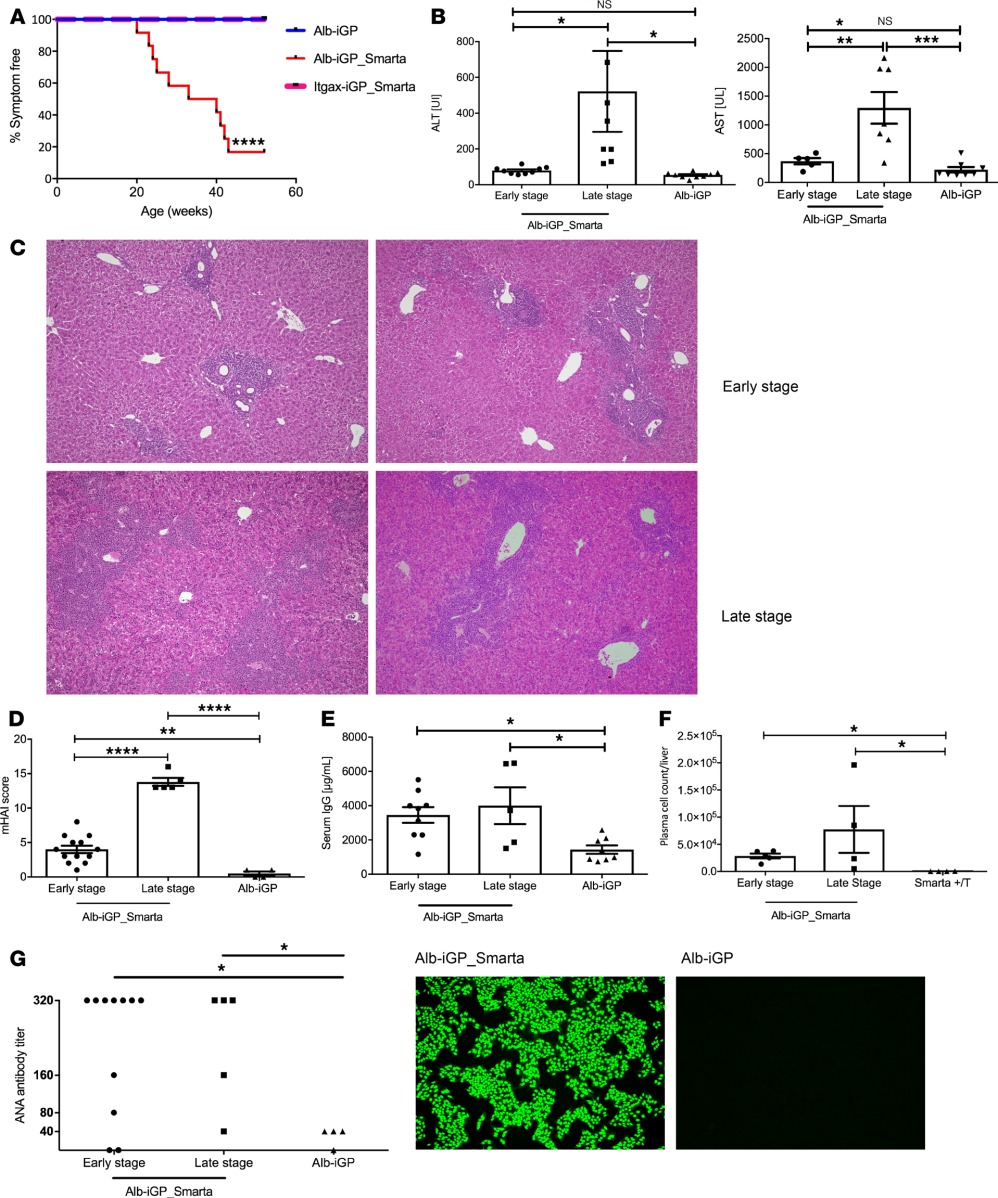
Spontaneous development of AIH features in Alb-iGP_Smarta mice. (**A**) Spontaneous development of sickness symptoms, shown as age-dependently reduced percentage of symptom-free Alb-iGP_Smarta mice (red line; *n* = 12), compared with Alb-iGP control mice (blue line; *n* = 13) and Itgax-iGP_Smarta control mice (pink line; *n* = 10). (**B**) Serum ALT and AST (each U/l; *n* = 8–9) in Alb-iGP_Smarta mice at early or late disease stage, and Alb-iGP control mice. (**C**) Representative histology of 2 Alb-iGP_Smarta livers in early disease stage, showing periportal infiltrates (top), or late disease stage (bottom), showing periportal and interface hepatitis with mainly lymphocytic infiltrates (original magnification, ×100). (**D**) The mHAI score (*n* = 4–13) of Alb-iGP_Smarta livers (early and late disease) and Alb-iGP control livers. (**E**) Serum IgG levels (g/ml; *n* = 5–9). (**F**) Hepatic CD19^+^CD138^+^ plasma cells (*n* = 4–5). (**G**) Antinuclear antibody (ANA) titers (*n* = 4–11) and representative fluorographs. Data are shown as the mean ± SEM. *P < 0.05; **P < 0.01; ***P < 0.001; ****P < 0.0001 (**A**, Mantel-Cox; **B**, **D**, and **E**, ANOVA; **F**, Fisher’s exact test).

To address whether the conditional expression of GP in hepatocytes and the presence of GP-specific Smarta cells resulted in autoimmune disease, we monitored Alb-iGP_Smarta mice over time and, as control, Itgax-iGP_Smarta mice and Alb-iGP mice that both lacked GP-specific Smarta cells. Whereas Itgax-iGP_Smarta and Alb-iGP mice remained healthy over an observation period of 1 year, Alb-iGP_Smarta mice that were replete with GP-reactive T cells developed spontaneous AIH-like disease (Figure 2), which could be classified into 2 distinct disease stages. Young Alb-iGP_Smarta mice (4–20 weeks; “early stage”) presented without apparent disease symptoms (Figure 2A) and had normal serum transaminases (Figure 2B) but manifested portal infiltration in liver histology (Figure 2C, top). Beginning at an age of 20 weeks (“late stage”), Alb-iGP_Smarta mice gradually manifested spontaneous sickness symptoms, such as reduced mobility, moderate piloerection, and altered facial expression or posture (Figure 2A). At 30 weeks of age, 50% of the mice manifested disease symptoms. This phenotypical sickness was always associated with significantly elevated serum transaminase levels (Figure 2B; late stage; alanine aminotransferase [ALT], *P* = 0.0312; aspartate aminotransferase [AST], *P* = 0.0006). The predominant histological image of these apparently sick mice was that of portal lymphocytic infiltrates with interface hepatitis (Figure 2C; bottom). Accordingly, histological activity, scored by a pathologist using the modified histologic activity index (mHAI) score, was significantly elevated already in the early stage in Alb-iGP_Smarta mice, as compared with Alb-iGP control mice (*P* = 0.0048), and significantly increased further in Alb-iGP_Smarta mice in late stage (Figure 2D; *P* = 0.0001). Moreover, Alb-iGP_Smarta mice had elevated serum IgG levels, both in early and late stage (Figure 2E; *P* = 0.0285 and *P* = 0.0174), and accumulated plasma cells in the liver (Figure 2F; *P* = 0.0159 and *P* = 0.0286). Accordingly, Alb-iGP_Smarta mice developed antinuclear autoantibodies (ANAs) at relevant titers already in the early stage (Figure 2G). The penetrance of typical AIH features in early and late stage is summarized in Table 1.

### Pathogenic maturation of GP-reactive CD4^+^ effector T cells within the livers of GP peptide–expressing mice.

**Figure 3:**
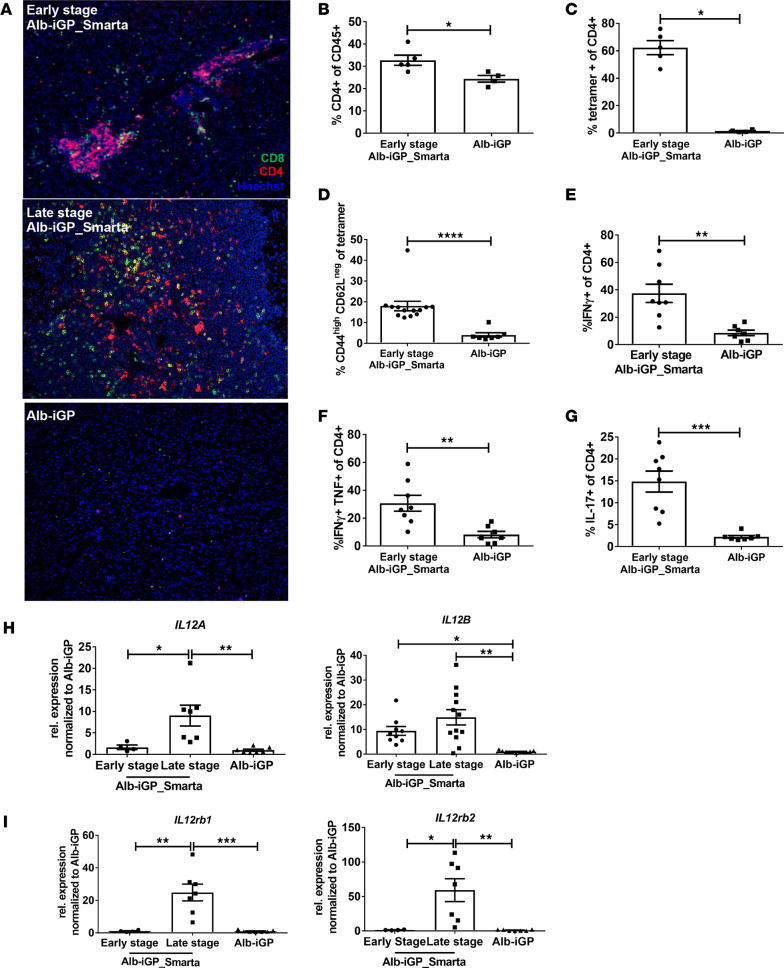
Characterization of liver-infiltrating T cells in Alb-iGP_Smarta mice. (**A**) Immunofluorescence of liver sections revealing infiltration of CD4 (red) and CD8 cells (green) in Alb-iGP_Smarta (top, early stage; middle, late stage) or Alb-iGP control mice (bottom). Nuclei are stained in blue (original magnification, ×100). (**B**) Percentage of liver-infiltrating CD4^+^ T cells in Alb-iGP Smarta and Alb-iGP control mice (*n* = 4–5). (**C**) Percentage of GP tetramer-binding CD4^+^ T cells in livers of Alb-iGP_Smarta and Alb-iGP control mice (*n* = 4–5). (**D**) Percentage of liver-infiltrating CD4^+^ T cells with activated/memory phenotype in Alb-iGP_Smarta and Smarta control mice (*n* = 7–13). (**E**) Frequencies of IFN-γ–producing CD4^+^ T cells, (**F**) TNF and IFN-γ coproducing CD4^+^ T cells, and (**G**) IL-17–producing CD4^+^ T cells in livers of Alb-iGP_Smarta and Alb-iGP control mice (*n* = 4–8). (**H**) Hepatic gene expression of *IL12A* and *IL12B* and (**I**) *IL12rb1* and *IL12rb2* in Alb-iGP_Smarta mice in early or late stage and Alb-iGP control mice (*n* = 4–12). Data are shown as the mean ± SEM. *P < 0.05; **P < 0.01; ***P < 0.001; ****P < 0.0001 (**B–E**, Mann-Whitney; **F–I**, ANOVA).

Histological analysis suggested that hepatic infiltrates in Alb-iGP_Smarta mice, both in early and late disease stage, consisted mainly of CD4^+^ T cells (Figure 3A, red); however, some CD8^+^ T cells (Figure 3A, green) were also found, notably in the late stage. Since the development of AIH-like disease in Alb-iGP_Smarta mice seemed to be driven by activation of autoreactive CD4^+^ T cells, we analyzed the liver-infiltrating T cells in the early stage. A significant increase of CD4^+^ T cells numbers in livers of Alb-iGP_Smarta mice was confirmed by flow cytometry as compared with that in Alb-iGP control mice that express GP in the liver but lack GP-specific T cells (Figure 3B). The majority of the liver-infiltrating CD4^+^ T cells recognized the I-A(b) LCMV GP66–77 tetramer (Figure 3C; the gating strategy and representative staining are shown in Supplemental Figure 1; supplemental material available online with this article; https://doi.org/10.1172/jci.insight.141462DS1) and displayed an activated effector or memory T cell phenotype (Figure 3D and Supplemental Figure 2A; *P* = 0.0005), as compared with Smarta control mice that feature GP-specific T cells but lack GP expression in the liver. The liver-infiltrating CD4^+^ T cells in Alb-iGP_Smarta mice were dominated by Th1 cells producing IFN-γ (37.5%, Figure 3E; *P* = 0.0012), which were significantly increased as compared with Alb-iGP control mice (8.6%). Interestingly, and in accordance with our recent findings in human AIH (36), the liver-infiltrating Th1 cells in Alb-iGP_Smarta mice were marked by coproduction of IFN-γ and TNF in liver (Figure 3F and Supplemental Figure 2B; *P* = 0.0012), indicating potent inflammatory activation. The fraction of IL-17 producers among the intrahepatic CD4^+^ T cells was also elevated in Alb-iGP_Smarta mice (14.8 vs. 2.2% in Alb-iGP controls; Figure 3G; *P* = 0.0003). Corresponding to the predominant Th1 phenotype of the intrahepatic CD4^+^ T cells, we found elevated expression of *Il12a* (Figure 3H; *P* = 0.0048) and *Il12b* (Figure 3H; *P* = 0.0003) in the livers of Alb-iGP_Smarta mice. Accordingly, expression of the IL-12 receptor genes *Il12rb1* (Figure 3I; *P* = 0.0002) and *Il12rb2* (Figure 3I; *P* = 0.0024) was also upregulated in the livers of Alb-iGP_Smarta mice.

**Figure 4:**
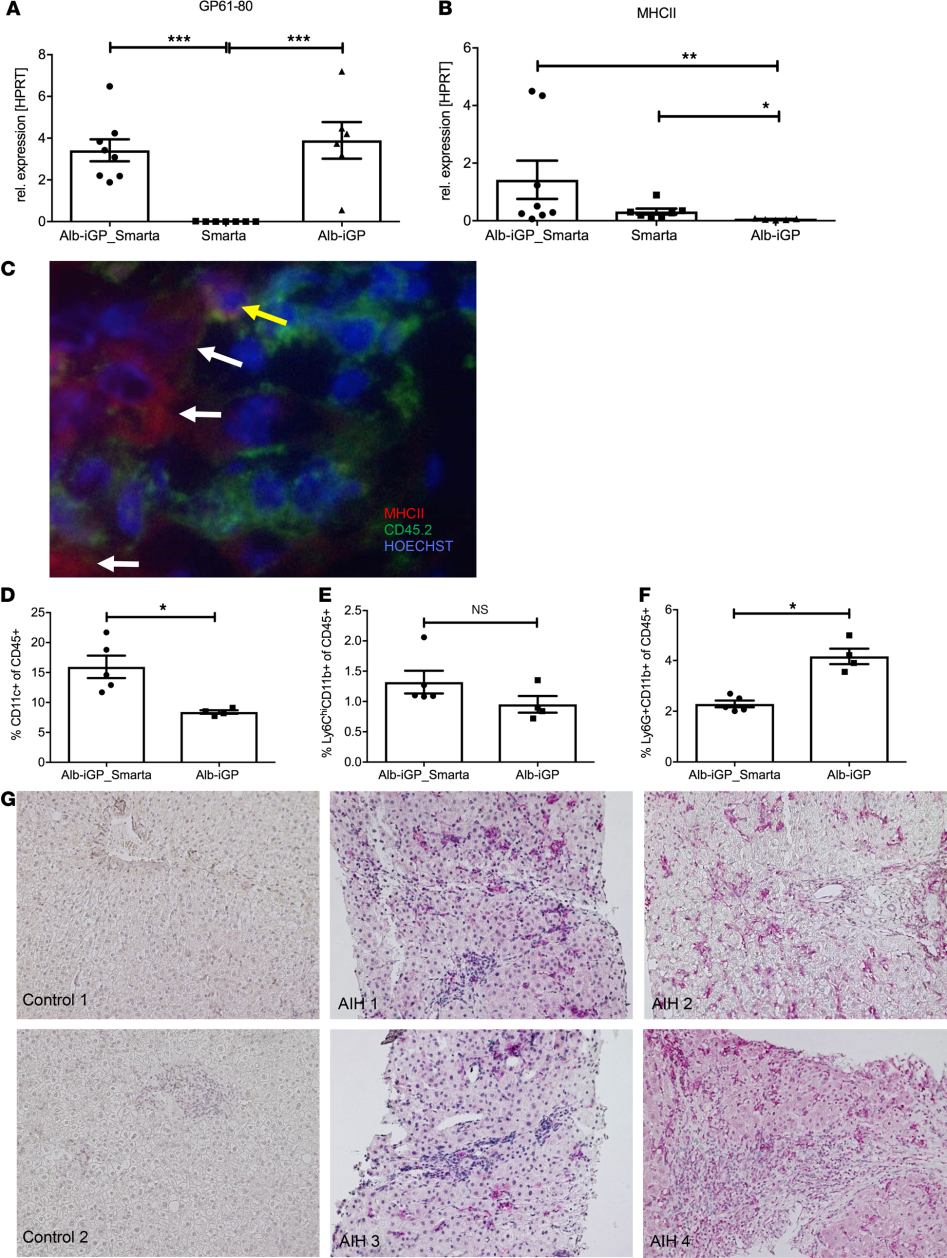
DCs as pathogenic drivers in Alb-iGP_Smarta mice and human AIH. Hepatocellular gene expression of GP (**A**) and I-A^b^ (**B**) in Alb-iGP_Smarta, Smarta, and Alb-iGP mice (*n* = 6–8). (**C**) Immunostaining of MHC II (red) and CD45 (green) in livers of Alb-iGP Smarta mice. Nuclei are stained in blue. The yellow arrow indicates a CD45^+^MHC II^+^ APC; the white arrows indicate CD45^–^MHC II^+^ hepatocytes (original magnification, ×400). (**D**) CD11c^+^ DCs, (**E**) Ly6C^hi^CD11b^+^ monocytes, and (**F**) Ly6G^+^CD11b^+^ neutrophils in livers of Alb-iGP Smarta mice at early stage as compared with Alb-iGP controls. (**G**) Histological analysis of liver biopsies from newly diagnosed, untreated patients with AIH, as compared with pseudo-healthy control tissue from subjects undergoing bariatric surgery (original magnification, ×100). CD11c^+^ DCs are stained in pink. Data are shown as the mean ± SEM. *P < 0.05; **P < 0.01; ***P < 0.001 (**A** and **B**, ANOVA; **D–F**, Mann-Whitney).

To understand how the inflammatory activation of the autoreactive CD4^+^ T cells occurred in the liver, we purified hepatocytes from Alb-iGP_Smarta mice or from Alb-iGP or Smarta control mice and analyzed the expression of the cognate GP peptide and of MHC II molecules in hepatocytes by quantitative RT-PCR. Expression of both the GP peptide (Figure 4A; *P* = 0.0002) and the MHC II molecule I-A^b^ (Figure 4B; *P* = 0.0023) was significantly upregulated in 8-week-old Alb-iGP_Smarta mice. To confirm this finding, we performed immunofluorescence staining of liver sections for MHC II and for CD45 to unequivocally distinguish CD45^–^ hepatocytes from CD45^+^ antigen-presenting cells (APCs). We found that some hepatocytes indeed did upregulate MHC II molecules (Figure 4C); however, the majority of hepatocytes did not show detectable levels of MHC II. Thus, hepatocytes presenting GP peptides at low level seemed to participate in the inflammatory activation of autoreactive CD4^+^ T cells in Alb-iGP_Smarta mice, but probably not as major drivers. Yet we also found many CD45^+^ MHC II–expressing APCs in the livers of these mice, which prompted us to investigate these cells further. In flow cytometry, we observed a significant increase of CD11c^+^ DCs in the livers of Alb-iGP_Smarta mice (Figure 4D; *P* = 0.0159), whereas Ly6C^hi^CD11b^+^ monocytes (Figure 4E; *P* = 0.1905) or Ly6G^+^CD11b^+^ granulocytes (Figure 4F; *P* = 0.0159) were not increased. To confirm that DCs might also drive tissue inflammation in human AIH, liver biopsies from 4 patients with active, newly diagnosed AIH before treatment were analyzed histologically (Figure 4G and Table 2). Indeed, in contrast to pseudo-healthy control tissue from 2 subjects undergoing bariatric surgery, CD11c^+^ DCs were abundantly present in hepatic infiltrates in all studied AIH samples (Figure 4G).

**Figure 5:**
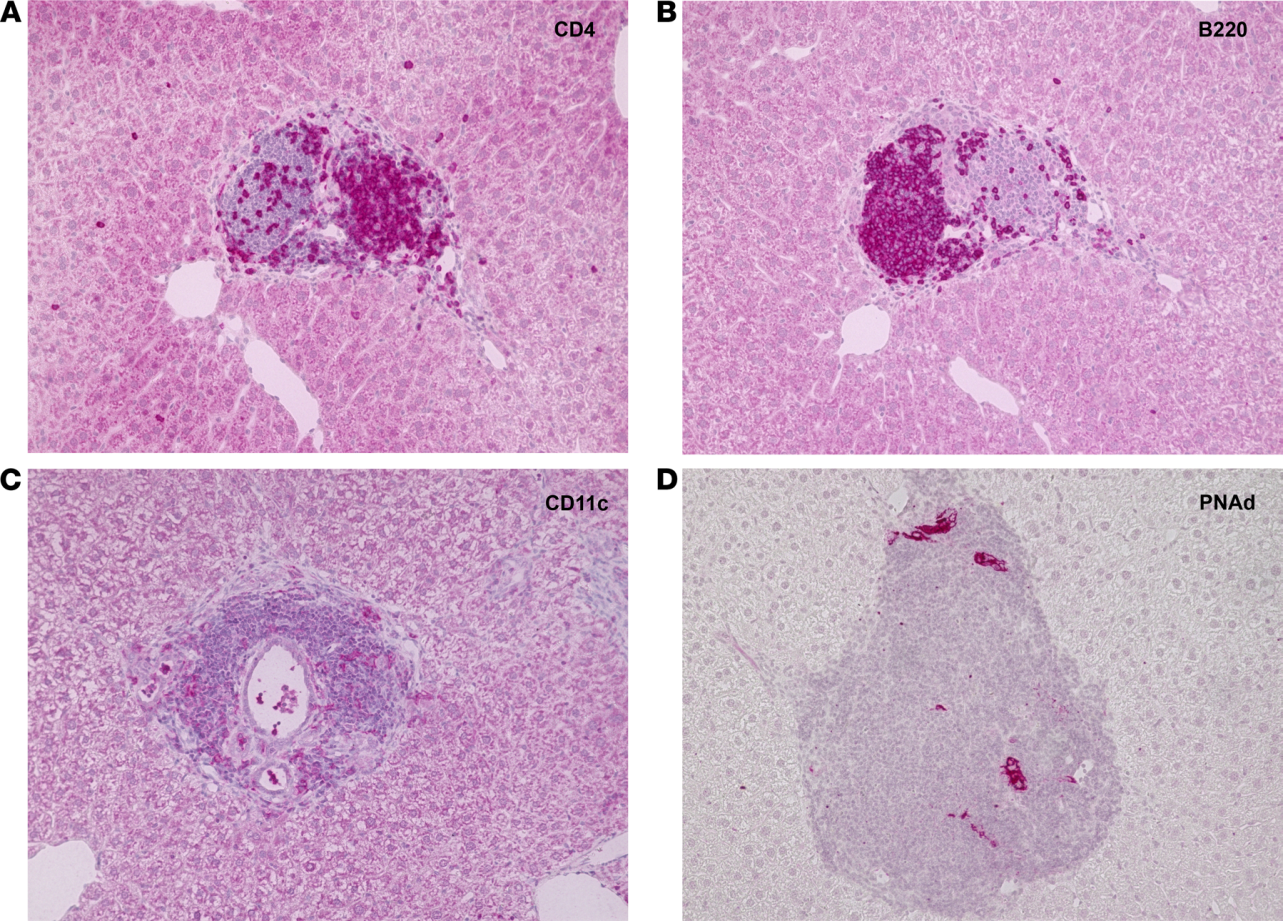
Histological characterization of portal ectopic lymphoid tissue in early disease livers of Alb-iGP_Smarta mice. Segregation of CD4^+^ T cells (**A**) and B220^+^ B cells (**B**) in separate periportal zones in consecutive sections of Alb-iGP_Smarta livers. (**C**) CD11c^+^ DC network in periportal infiltrate of Alb-iGP_Smarta liver. (**D**) PNAd^+^ high endothelial venules in periportal infiltrate of Alb-iGP_Smarta liver (original magnification, ×200).

The inflammatory activation of liver-infiltrating CD4^+^ T cells seemed to occur mainly in the portal tracts of Alb-iGP_Smarta mice, as suggested by the histological imaging of considerable periportal infiltrates in the early disease stage (Figure 2C and Table 1). We noted that these early stage periportal infiltrates often had the appearance of ELT. To confirm this unexpected finding, we performed additional immunohistochemical staining and found the characteristically segregated T and B cell zones in periportal infiltrates, which are typical for ELT (Figure 5, A and B). Moreover, we detected the typical network of DCs (Figure 5C) and presence of high endothelial venules (Figure 5D), confirming the formation of ELTs in the livers of Alb-iGP_Smarta mice at the early stage of AIH. Of note, these ELTs had been completely disaggregated in the late disease stage of Alb-iGP_Smarta mice, in which the ELT had apparently merged into the more extensive, less ordered and dispersed infiltrates of interface hepatitis. These findings suggested that hepatic ELTs, which normally remain undetected in preclinical human AIH, might be a relevant site of inflammatory activation of autoreactive T cells.

### Selective instability and reduced suppressor function in GP-reactive Tregs.

**Figure 6:**
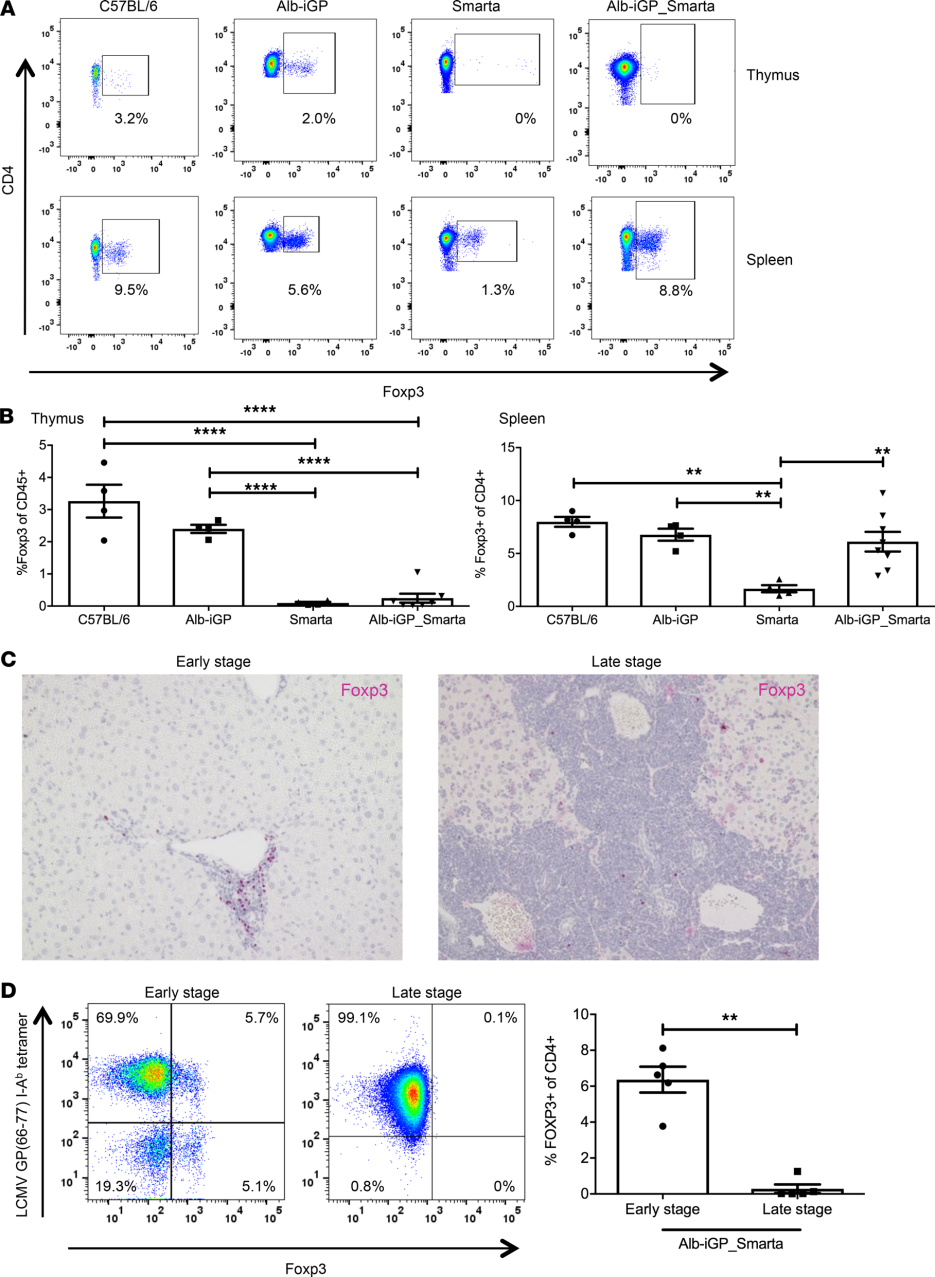
Quantification of Tregs in Alb-iGP_Smarta mice. (**A**) Representative flow cytometry dot plots of thymic and splenic Foxp3^+^ Tregs in C57BL/6, Alb-iGP, Smarta and Alb-iGP_Smarta mice. (**B**) Frequencies of thymic (left) and splenic (right) CD4^+^Foxp3^+^ Tregs in the respective mouse strains (*n* = 4–8). (**C**) Representative Foxp3 immunohistochemistry of Tregs in periportal infiltrates of Alb-iGP_Smarta liver in early disease (left) or late disease (right) (original magnification, ×200). (**D**) Flow cytometry of antigen-specific (tetramer^+^) and nonspecific (tetramer^–^) CD4^+^ Foxp3^+^ Tregs in Alb-iGP_Smarta liver in early disease (left) or late disease (right) and quantification of Treg numbers in early and late stage (*n* = 5). Data are shown as the mean ± SEM. **P < 0.01; ****P < 0.0001 (**B**, ANOVA; **D**, Mann-Whitney).

The question then was why autoreactive effector T cell activation was not restricted by Tregs. To elucidate the possible role of Tregs in AIH pathogenesis, we first analyzed thymic and peripheral Treg generation in Alb-iGP_Smarta mice (Figure 6A) and found that thymic Treg induction was negligible, whereas peripheral Treg induction was significantly increased in Alb-iGP_Smarta mice (6.1% splenic Tregs), as compared with Smarta mice that do not feature the GP61–80 autoepitope (1.8% splenic Tregs; Figure 6B; *P* = 0.068). As indicated by immunohistochemical staining of Alb-iGP_Smarta livers, Foxp3^+^ Tregs were present in the early disease stage but greatly reduced in the late disease stage (Figure 6C). This finding was confirmed by flow cytometry (Figure 6D; *P* = 0.0079). These findings indicated that the expansion of Smarta effector cells in late disease was associated with and probably enabled by greatly reduced Treg numbers.

**Figure 7:**
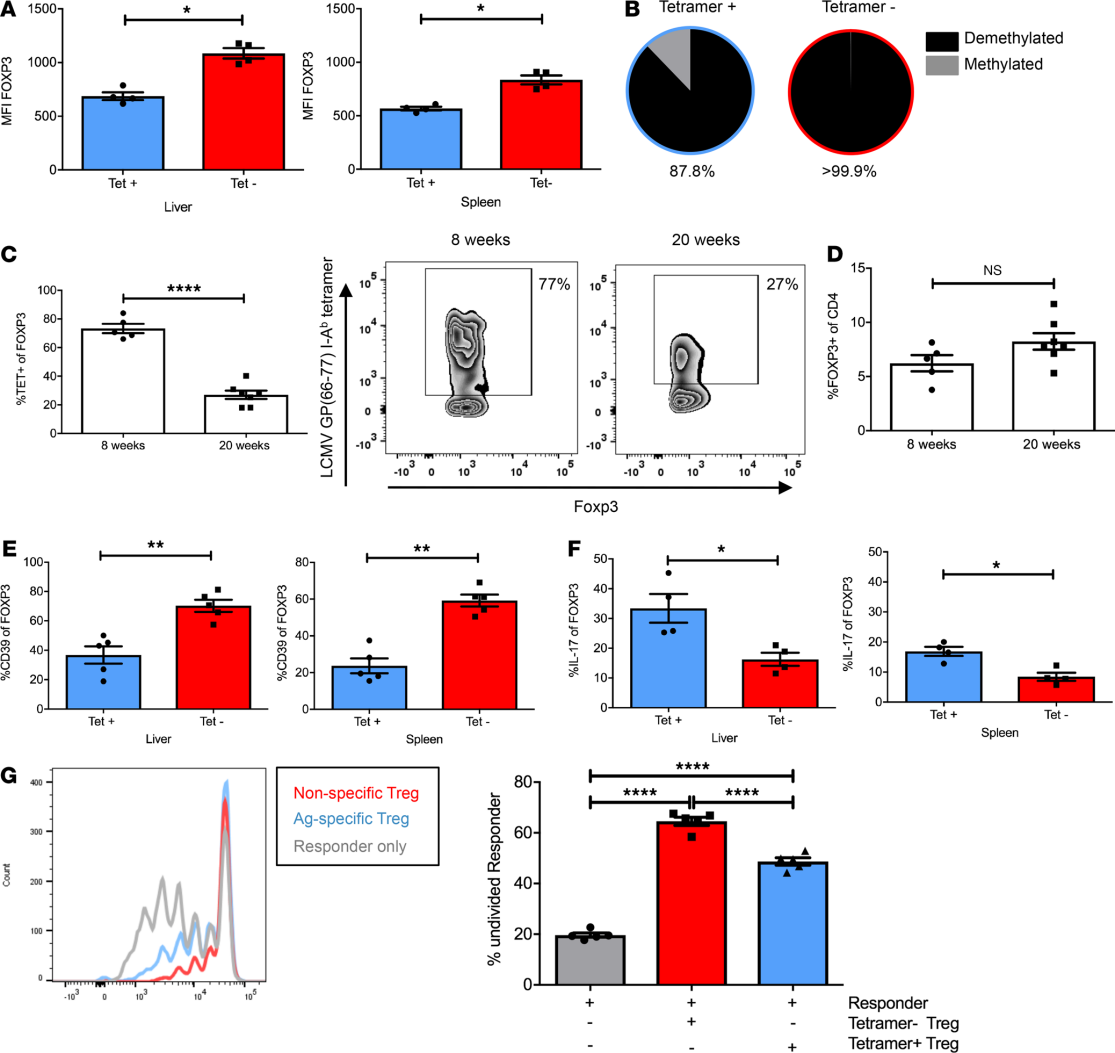
Functional characterization of Tregs in Alb-iGP_Smarta mice. (**A**) Selectively reduced Foxp3 MFI in tetramer-specific vs. nonspecific CD4^+^ Foxp3^+^ Tregs in livers and spleens of Alb-iGP_Smarta mice (*n* = 4). (**B**) Reduced CNS2 element demethylation at the Foxp3 gene locus in tetramer-specific vs. nonspecific splenic CD4^+^ Foxp3^+^ Tregs purified and pooled from 10 Alb-iGP_Smarta mice. (**C**) Age-dependent decline of tetramer-specific vs. nonspecific CD4^+^ Foxp3^+^ Tregs (*n* = 5–7). (**D**) No age-dependent change in overall Foxp3^+^ Treg frequency among CD4^+^ T cells (*n* = 5–7). (**E**) Selectively reduced percentage of CD39-expressing cells, and (**F**) selectively increased percentage of IL-17 producers among tetramer-specific vs. nonspecific CD4^+^Foxp3^+^ Tregs in livers and spleens of Alb-iGP_Smarta mice (*n* = 4). (**G**) Selectively reduced suppressive function of tetramer-specific (blue) vs. nonspecific (red) splenic CD4^+^Foxp3^+^ Tregs of Alb-iGP_Smarta mice (*n* = 5). Data are shown as the mean ± SEM. *P < 0.05; **P < 0.01; ****P < 0.0001 (**A**, **C**, and **D**, Mann-Whitney; **E**, ANOVA).

To better understand how this Treg reduction could have occurred, we analyzed the intrahepatic and splenic Foxp3^+^ Tregs in the early disease stage. Intriguingly, we found that the expression of Foxp3 was selectively reduced in the GP-specific Tregs (Figure 7A and Supplemental Figure 3A), as indicated by significantly decreased mean fluorescence intensity (MFI) of Foxp3 in comparison to the Foxp3 MFI of nonspecific Tregs (liver, 686.8 vs. 1086, *P* < 0.0001; spleen, 568 vs. 835; *P* = 0.0286). Note that stable maintenance of Foxp3 expression is important for Treg function, and reduced Foxp3 expression can indicate Treg instability and reduced Treg function (33, 37, 38). Stable Foxp3 expression is mainly maintained by epigenetic modification of the *Foxp3* gene locus and, in particular, by full demethylation of the conserved noncoding DNA sequence element 2 (CNS2) (also known as the Treg-specific demethylated region) (38–40). Thus, to confirm that GP-specific Tregs exhibited reduced stability, we sorted pooled antigen-specific or nonspecific splenic Tregs of 10 mice, based on tetramer-binding and Foxp3-gfp reporter activity, taking advantage of Foxp3-gfp reporter mice (41) bred onto Alb-iGP_Smarta background. Demethylation analysis of the CNS2 element revealed that the GP-specific Tregs showed a considerably reduced degree of *Foxp3* gene demethylation, as compared with nonspecific Tregs (88% vs. 100%; Figure 7B). As a consequence of the selective instability of antigen-specific Tregs, their frequency declined significantly between week 8 of age, when about 75% of all Tregs were antigen specific, and week 20, when only about 30% antigen-specific Tregs remained (Figure 7C; *P* < 0.0001). Note that, in contrast, the overall Treg frequency did not change significantly between weeks 8 and 20 (Figure 7D).

We tested whether the instability of GP-specific Tregs was associated with reduced expression of the functional molecule CD39 (Figure 7E). We found indeed that CD39 was reduced in splenic (*P* = 0.0079) as well as intrahepatic GP-specific Tregs (*P* = 0.0079), as compared with nonspecific Tregs. Moreover, a significantly increased proportion of the splenic as well as intrahepatic GP-specific Tregs produced IL-17 (Figure 7F and Supplemental Figure 3B), as compared with nonspecific intrahepatic Tregs (liver, 33.4% vs. 16.3%; *P* = 0.0286; spleen, 16.8 vs. 8.4; *P* = 0.0286). Note that such a Th17-like phenotype in Tregs indicates a reversible loss of suppressor activity (42). Moreover, again using Foxp3-gfp reporter activity to sort GP-specific or nonspecific Tregs, we could directly compare their suppressive capacity in an in vitro suppression assay. As can be seen in Figure 7G, GP-specific Tregs were significantly less functional as suppressors compared with nonspecific Tregs (*P* < 0.0001). These findings demonstrated that the GP-specific Tregs were indeed selectively impaired in Alb-iGP_Smarta mice.

## Discussion

The mechanisms that allow autoreactive T cells to defy immune regulation and to cause self-sustained autoimmune diseases are not fully understood. In particular, it is unclear how the loss of tolerance to tissue-restricted antigens might occur at immune-privileged sites such as the liver. Here, we describe a mouse model of spontaneous CD4^+^ T cell–driven AIH, which allowed us to address several key questions related to the break of tolerance in the liver and the pathogenesis of AIH.

### What is the role of thymic tolerance in autoimmune pathogenesis?

In the thymus, the medullary thymic epithelial cells can induce tolerance to many self-antigens, including liver antigens. They can do this because these cells feature several transcriptional programs coordinated by the transcription factors Aire, Fezf2, and Prdm1, which facilitate the ectopic expression of a wide array of tissue-restricted antigens (43–45). However, different self-antigens vary in their degree of thymic representation, and these representation patterns have been demonstrated to determine the fate of the immature T cells in the thymus. Indeed, Malhotra et al. showed that (a) peptides that are uniformly presented by thymic antigen-presenting cells produce clonal deletion, (b) peptides with limited thymic expression induce partial clonal deletion and thymic generation of Tregs, and (c) peptides that are excluded from thymus are ignored (46). Thus, autoreactive T cells recognizing tissue-restricted autoantigens, which are rarely expressed in the thymus, are prone to escape clonal deletion. Our model confirmed that assumption by showing that GP-specific CD4^+^ T cells were not deleted in the thymi of Alb-iGP_Smarta mice, due to absent thymic GP expression (Figure 1); in contrast, the high degree of thymic GP expression in Itgax-iGP_Smarta mice was associated with near-complete deletion of autoreactive CD4^+^ T cells. Importantly, as a consequence of the absent deletion in the thymi of Alb-iGP_Smarta mice, autoreactive T cells abounded in the periphery. Upon antigen recognition in the liver, the autoreactive effector cells were activated, enabling spontaneous development of the typical disease features of AIH (Table 1 and Figure 2).

### What drives the activation of autoreactive effector T cells?

We found that the pathogenic activation of the previously ignorant GP-reactive CD4^+^ T cells in Alb-iGP_Smarta mice seemed to require several weeks of subclinical autoimmunity before the spontaneous clinical manifestation of self-sustained autoimmune disease. Of note, this pathogenic maturation seemed to occur in the liver. Indeed, we found that at least some hepatocytes had upregulated MHC II expression (Figure 4, B and C), which hence might have contributed to the activation of GP-reactive CD4^+^ T cells. However, we also observed a substantial expansion of DCs in the livers in young Alb-iGP_Smarta mice already (Figure 4D), indicating that DCs, being potent professional APCs, most likely were the major drivers of the pathogenic activation of previously ignorant autoreactive CD4^+^ effector T cells. Importantly, we also found a substantial expansion of CD11c^+^ DCs in human treatment-naive AIH (Figure 4G). In mice, we often observed the transient formation of portal ELT in Alb-iGP_Smarta mice (Figure 5). ELTs are organized structures of T cells, together with antigen-presenting cells, that are suspected to boost autoimmune pathogenesis (20). For yet unclear reasons, these highly ordered ectopic lymphoid structures seemed to disaggregate in later disease. Whether similar, transient structures might also be involved in preclinical stages of human AIH is currently not clear, as human AIH is usually diagnosed only in the clinical disease stage, in which these structures were also absent in mice. Yet, irrespective of the microanatomical specifics, our model revealed that intrahepatic expansion of professional APCs seems to be a characteristic of both human and murine AIH, providing a stimulation platform for the inflammatory activation of autoreactive T cells locally in the liver. This pathogenic activation seemed to involve IL-12 (Figure 3, H and I), which is the major driver of Th1-like effector T cell responses. Accordingly, the pathogenic activation of GP-reactive CD4^+^ T cells in mice was marked by coproduction of the classical Th1 cytokines IFN-γ and TNF (Figure 3F). Of note, we found a similar dominance of IFN-γ and TNF coproducers among the liver-infiltrating CD4^+^ T cells in human AIH (36), indicating that this cytokine signature might be relevant for AIH pathogenesis. Our findings reemphasize the previously reported pathogenic role of IL-12 in murine AIH (19). Therefore, intrahepatically expanded DCs and the DC-derived cytokine IL-12 might be relevant treatment targets in AIH that should be explored in future studies.

### What is the role of Tregs in the break of tolerance to liver antigens?

Tregs have the potential to suppress the activation of autoreactive effector T cells that have escaped clonal deletion, most notably when they recognize the same autoantigen as the effectors (6, 24). Importantly, GP-reactive Tregs were present in early stage AIH livers of Alb-iGP_Smarta mice (Figure 6). This is reminiscent of human AIH, in which several groups have reported that Tregs do accumulate in inflamed AIH livers (27–29). Yet, both the human and murine intrahepatic Tregs do not seem to be effective in suppressing effector T cells in the inflamed liver. Here, we show that this functional deficit might be explained by Treg instability and plasticity, which selectively occurred in autoantigen-specific but not in nonspecific Tregs. Importantly, this instability produced a significant reduction of the antigen-specific Tregs during the preclinical stage between week 8 and week 20 of age, whereas nonspecific Tregs remained stable (Figure 7, C and D). Treg instability and plasticity was marked by reduced Foxp3 expression, reduced demethylation of the CNS2 element at the *Foxp3* gene locus, reduced expression of the functional marker CD39, and production of IL-17 (Figure 7, E and F). Of note, these antigen-specific Tregs exhibited reduced suppressive activity, as compared with nonspecific Tregs (Figure 7G). The precise mechanisms leading to Treg instability and plasticity are not clear; however, these phenomena have been linked to autoimmune disorders (32, 33). Under the influence of inflammatory cytokines, Tregs can convert into Th-like cells that also produce effector cytokines while maintaining Foxp3 expression (33, 47–49). Here we found that Tregs acquired Th17-like features, an adaptation that has been reported to result in reduced suppressive potential under inflammatory conditions (42, 47). Thus far, it is not clear whether Th17-like Tregs are a stable cell type or represent an intermediate state in a conversion process (50, 51). Nonetheless, our finding is important, as it demonstrates that AIH pathogenesis does not require a generalized Treg defect, as had been proposed before (26). In contrast, Treg dysfunction in AIH seems to occur selectively in autoantigen-specific Tregs. These antigen-specific Tregs might have been prone to become instable due to their peripheral, not thymic, origin (see Figure 6), as peripherally induced Tregs usually feature lower CNS2 demethylation and reduced stability as compared with thymus-induced Tregs (38, 39). Moreover, we think that antigen-specific Treg instability was most likely an adaptation to inflammatory conditions in the liver (52). Note that we have previously identified IL-12 as an important mediator of intrahepatic Treg dysfunction under inflammatory conditions (31). Thus, we found it most likely that the here-identified hepatic upregulation of IL-12 not only had driven the pathogenic effector cell activation, as explained above, but also the observed instability of the antigen-specific Tregs.

Due to its similarities to human AIH, we think that this AIH model provides a valuable paradigm to uncover immune mechanisms of the thus far elusive pathogenesis of AIH and to identify potential treatment targets. Yet it seems to be less suited for treatment studies, given its variable disease onset and rapid progression. Moreover, the model does not show the sexual dimorphism in disease prevalence that is found in human disease. However, as an explanation for female preponderance of autoimmunity, it has been suggested that female sex hormones repress AIRE expression in the thymus, allowing more autoreactive T cells to escape thymic selection (53). It is thus conceivable that our model did not manifest a sexual dimorphism, because thymic GP expression was virtually absent in both sexes and thus not influenced by sex hormones.

In summary, our model revealed that AIH seems to be driven by activation of previously ignorant autoreactive CD4^+^ effector T cells and differentiation toward IFN-γ and TNF coproducers. Effector cell activation and differentiation seemed to be induced locally in the liver by an expanded population of intrahepatic DCs. Moreover, the autoantigen-specific Tregs in the inflamed liver, but not the nonspecific Tregs, selectively manifested increased plasticity and instability, suggesting that Treg dysfunction in AIH is not generalized, but restricted to autoreactive Tregs. Thus, autoimmune pathogenesis in an immune-privileged organ, such as the liver, seems to require both insufficient thymic deletion of autoreactive T cells and maladaptive peripheral regulation caused by instability of autoantigen-specific Tregs. Therefore, the success of Treg-based treatment attempts for autoimmune diseases affecting immune-privileged organs might depend on additional strategies to stabilize antigen-specific Treg phenotype and function.

## Methods

### Generation of invariant chain GP mice.

The targeting vector ROSA26STOP*IiMOG (34), a gift from Ari Waisman (University Medicine Mainz, Mainz, Germany), was used to generate the targeting vector ROSA26STOP*iGP by replacement of the MOG peptide encoding gene sequence with a gene sequence encoding the immunodominant GP peptide of LCMV. To that end, we generated an inducible mutant invariant chain with GP peptide (iGP) by assembly PCR on the liMOG template cDNA (34), replacing the coding sequence of the MOG peptide with that of GP61–80 peptide in the correct reading frame. This was done by amplifying 2 fragments of either the 5′ or the 3′ part of the invariant chain sequence using 1 of the 2 external primers li-fwd (5′-GGATCTGACATGGTAAGTAA-3′) and Ii-rev (5′-CGTATAGCATACATTATACG-3′), in conjunction with 1 of 2 internal primers that were used to replace the MOG sequence with the GP sequence: (GP-fwd, 5′-CCCCGACATCTACAAGGGCGTGTACCAGTTCAAGTCCGTGGAGTTCGACGATAACATGCTCCTTGGGCC-3′, and GP-rev, 5′-cggacttgaactggtacacgcccttgtagatgtcggggccgttcaggcccttcatgcgaaggctctcca-3′). The 2 fragments were assembled by PCR using the external primers li-fwd and li-rev to obtain 1 iGP minigene (886 bp) introducing 1 AscI restriction site to the 5′ end and 1 FseI restriction site to the 3′ end. The obtained iGP minigene was amplified after cloning into the pGEM-T Easy vector (Promega). The original targeting vector ROSA26STOP*IiMOG, as well as the iGP minigene, were restricted with AscI and FseI, followed by ligation of the 2 resulting gene fragments of 750 bp and 14.2 kb. The targeting vector was linearized with PvuI and electroporated into ES cells, which were cultivated as described previously (54). Homologous recombinants were identified by Southern blot analysis using a 700 bp genomic EcoRI-PacI fragment after EcoRI digest (data not shown). Chimeras were generated from 2 homologous recombinant clones by injection into blastocysts. Germline transmission was confirmed by Southern blot analysis after EcoRI digest using a 1 kb SacII-XbaI fragment (probe 1) from pROSA26-1. ES cell culture, targeting, and implantation were performed by Irm Hermans-Borgmeyer (Center for Molecular Neurobiology Hamburg, Hamburg, Germany).

### Animal experimentation.

To achieve conditional expression of the GP peptide in hepatocytes, iGP mice were crossed with Smarta mice (35), and the resulting iGP_Smarta mice were then further bred with Alb-Cre mice to create Alb-iGP_Smarta mice that feature both hepatocellular expression of the GP61–80 peptide and CD4^+^ T cells recognizing the GP61–80 peptide sequence. Accordingly, to obtain Itgax-iGP_Smarta mice with GP61–80 expression mainly restricted to DCs, iGP_Smarta mice were bred with Itgax-Cre mice. As indicated, Foxp3gfp.KI reporter mice (41) were backcrossed onto the Alb-iGP_Smarta background. All mice were bred and kept under specific pathogen–free conditions with 12-hour-light/dark cycles in the animal facility of the University Medical Center Hamburg-Eppendorf, with access to water and standard chow diet (1318 rodent diet, Altromin) ad libitum. The mice were monitored daily for sickness symptoms, according to general appearance, body condition, posture, facial expression, and mobility for a period of up to 60 weeks. Clinical and behavioral humane endpoints were applied to minimize any harm. All animals received humane care.

### Cell isolation.

Primary cells were isolated from thymus, spleen, or liver. In brief, organs were passed through a 100 μm cell strainer to obtain single-cell suspensions. For isolation of liver nonparenchymal cells, the liver cell suspension was further purified by density gradient centrifugation as described previously (55). Tregs were isolated from spleens of Foxp3gfp.KI reporter mice crossed onto a Alb-iGP_Smarta background by fluorescence-based flow cytometrical sorting with a BD Aria III (BD Biosciences). These cells were then either subjected to DNA extraction with the DNeasy blood and tissue kit (Qiagen), followed by determination of the Foxp3 gene locus demethylation at Epiontis GmbH, or used in functional suppression assays described below. Hepatocytes were isolated as described previously (56).

### Flow cytometry.

Single-cell suspensions were stained with PacificOrange (Life Technologies) for dead cell exclusion and further stained for CD45 (clone 30-F11), CD4 (clone RM4-5), CD62L (clone MEL-14), CD39 (clone Duha59), CD19 (clone 1D3), CD138 (clone 281-2), TNF (clone MP6-XT22), and IL-17 (clone TC11-18H10.1) (all from BioLegend); CD8 (clone 53-6.7), CD44 (clone IM7), and IFN-γ (clone XMG1.2) (all from BD Bioscience); or Foxp3 (clone FJK-16S) (Thermo Fisher Scientific) using fluorochrome-labeled antibodies. As indicated, cells were also stained with APC-conjugated I-A(b) LCMV GP 66–77 tetramer, which was obtained from the NIH Tetramer Core Facility. The FoxP3 staining buffer set (Thermo Fisher Scientific) was used for Foxp3 and intracellular cytokine staining following restimulation in vitro with PMA/Ionomycin (MilliporeSigma) for 5 hours in the presence of the Golgi inhibitor GolgiPlug (BD Biosciences).

### Suppression assay.

In vitro suppression assays were performed as described previously (57). Briefly, purified antigen-specific and nonspecific Foxp3^+^ Tregs were sorted from Foxp3gfp.KI reporter mice on a Alb-iGP_Smarta background based on binding of APC-conjugated I-A(b) LCMV GP 66–77 tetramer and gfp reporter signals. Tregs were cocultured with splenic CD4^+^CD25^–^ non-Tregs from C57BL/6 mice stained with the CellTrace Violet Cell Proliferation Kit (Life Technologies) according to the manufacturer’s instructions before the cell culture. After stimulation in the presence of 1 μg/ml anti-CD3 (145-2C11) and 1 μg/ml anti-CD28 (37.51) antibodies (BioLegend) for 3 days in IMDM/10%FCS (Gibco), T cell proliferation was assessed by flow cytometry.

### Gene expression analysis.

For quantitative gene expression analysis, RNA was extracted from liver or thymus tissue with the NucleoSpin RNA kit (Macherey-Nagel) and reverse transcribed with the High Capacity cDNA Reverse Transcriptase Kit (Thermo Fisher Scientific) according to the manufacturer’s protocols. The cDNA was then subjected to real-time quantitative PCR using the Powerup SYBR green master mix (Thermo Fisher Scientific). GP target gene expression was detected using the primers CD74-fw (5′-CACTACTGCTTACTTCCTGT-3′) and GP-rev (5′-TCCACGGACTTGAACTGGTA-3′) (Eurofins), and normalized to mHprt house keeper levels using the following primers by employing the ΔΔCt method: mHprt-fw, 5′-GTTGGATACAGGCCAGACTTTGTTG-3′, and mHprt-rev, 5′-CTAATTTTACTGGCAACATCAACAG-3′ (Eurofins).

Alternatively, the following TaqMan Gene Expression Assays (Thermo Fisher Scientific) were used: *H2-Ab1*, Mm00439216_m1; *ll12a*, Mm00434169_m1; *Il12b*, Mm01288989_m1; *IL12rb1,* Mm00434189_m1; and *Il12rb2*, Mm00434200_m1.

### Immunohistology and fluorescence microscopy.

For analysis of liver inflammation, sections from in situ perfused and paraffin-fixed mouse livers were stained with hematoxylin and eosin (Roth) and scored by our pathologist in a blinded fashion by applying the mHAI score (58). To visualize CD4^+^ T cells, Foxp3^+^ Tregs, B220^+^ B cells, CD11c^+^ DCs (human and mouse), and PNAd^+^ high endothelial venules, the ZytoChem Plus AP Polymer Kit (POLAP Kit, Zytomed Systems) and the respective antibodies (PNAd antibody from BioLegend [MECA-79], CD11c from Invitrogen [PAS-79537], and all others from Thermo Fisher Scientific [4SM95, FJK-16S, RA3-6B2, M5/114.15.2]) were used. Aceton-fixed cryosections of snap-frozen livers were stained directly with fluorochrome-labeled antibodies against CD4 (clone GK1.5), CD8 (clone 53-6.7), and CD45 (clone 104) (BioLegend) or with rat anti-mouse MHC II (Invitrogen), followed by incubation with goat anti-rat IgG AF546 (A11081, Invitrogen), and counterstained with the nuclear dye Hoechst 33258.

### ANA detection.

ANAs were detected by indirect immunofluorescence using BIOCHIP slides (EUROIMMUN) loaded with human Hep2 cells, which were incubated with serial dilutions of mouse sera. Specific serum autoantibody binding was then visualized by immunofluorescence microscopy.

### Serology.

To assess liver damage, serum ALT and AST levels were measured at the Institute of Experimental Immunology and Hepatology, University Medical Centre Hamburg-Eppendorf, using a COBAS Mira System (Roche Diagnostic). IgG serum titers were measured with the IgG mouse ELISA Kit (Abcam). Serum cytokines were analyzed with the Milliplex MAP mouse cytokine/chemokine kit (MilliporeSigma) according to the manufacturer’s instructions.

### Statistics.

For statistical analysis of 2 groups, the Mann-Whitney test was applied. For comparison of more groups, the 1-way ANOVA test followed by Tukey′s post test was performed. Significance of ANA data was calculated with Fisher’s exact test with a cut-off titer of 1:80. Disease incidence was analyzed using the log-rank (Mantel-Cox) test. *P* < 0.05 was considered significant. Results in graphs are displayed as mean ± SEM.

### Study approval.

Animal studies have been approved by the review board of the state of Hamburg, Germany. The use of human liver specimens for histological analysis was approved by the responsible ethics committee of the “Landesärztekammer Hamburg” (reference no. PV3912), and all participants gave written informed consent. All animal experiments were carried out in accordance with the principles of the Basel Declaration and the European Directive 2010/63/EU.

## Author contributions

MP, AC, and JH conceived and designed the study. MP, LS, DL, DK, ALM, FAS, TP, M. Schakat, SW, and AC performed experimental work and data analysis. MP, AC, and JH drafted the manuscript. AWL, CWN, DS, CS, and M. Sebode provided technical or material support. JH acquired funding. All authors critically revised and approved the manuscript and accepted accountability.

## Supplementary Material

Supplemental data

## Figures and Tables

**Table 2:**
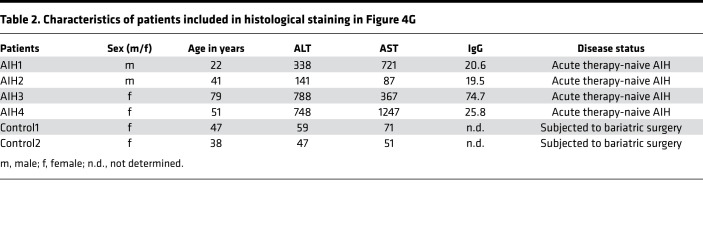
Characteristics of patients included in histological staining in Figure 4G

**Table 1:**
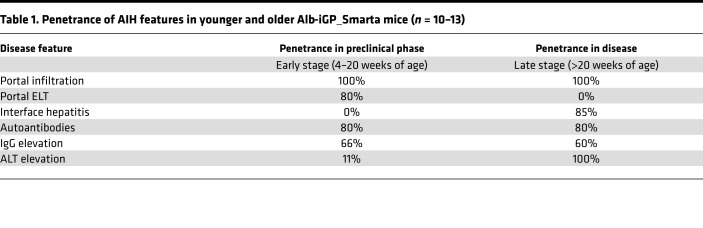
Penetrance of AIH features in younger and older Alb-iGP_Smarta mice (*n* = 10–13)

## Version 1 02/18/2021

In-Press Preview

## Version 2 03/22/2021

Electronic publication
