# Supplementary material for: Failure of thymic deletion and instability of autoreactive Tregs drive autoimmunity in immune-privileged liver
Source: JCI Insight. 2021 Mar 22;6(6):e141462. doi: 10.1172/jci.insight.141462 (PMC8026180; doi:10.1172/jci.insight.141462)
Supplement: Supplemental data [file jciinsight-6-141462-s127.pdf]

Supplementary Figure 1

A

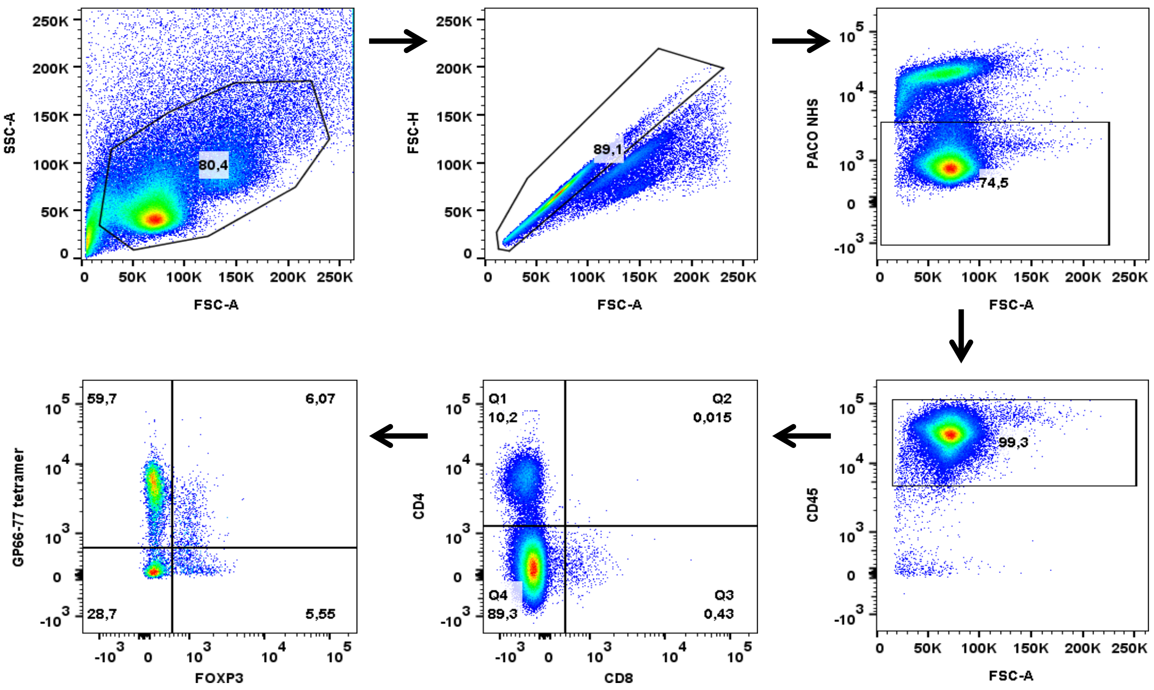

General gating strategy for flow cytometric analyses. In the first three steps, cellular debris (FSC vs. SSC), doublets (FSC-H vs. FSC-A) and dead cells (PACO-NHS vs. FSC-A) were excluded. Then, the residual CD45+ hematopoietic cells were further subgated according to the respective population of interest. In this example, hepatic antigen-specific tetramer+CD4+Foxp3+ Tregs of an Alb-iGP_Smarta mouse are shown.

Supplementary Figure 2

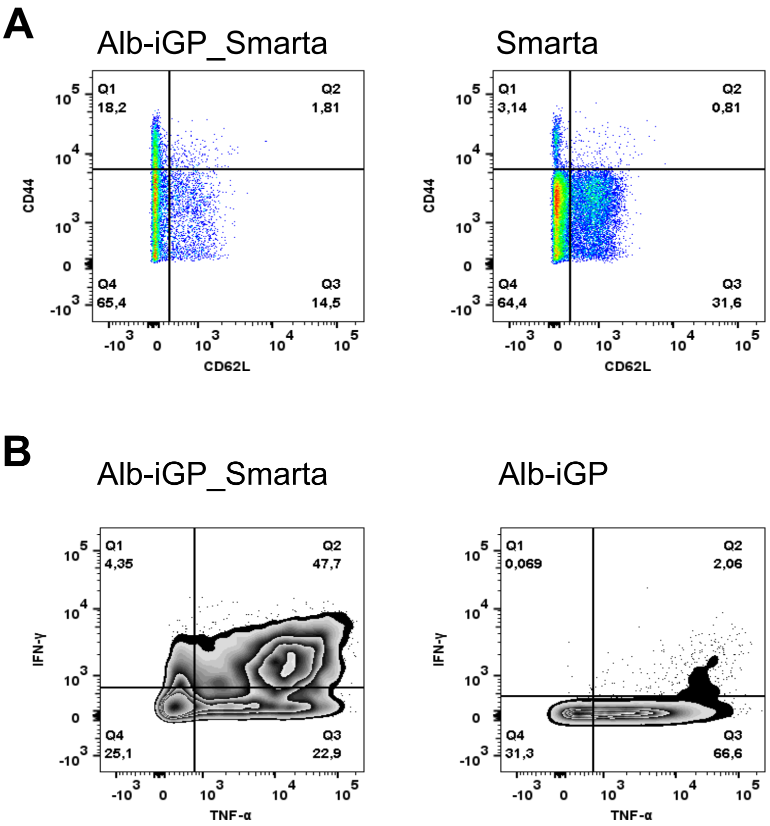

Characterization of liver-infiltrating CD4 T cells in Alb-iGP_Smarta mice. Representative dot plots showing (A) $CD44^{hi}$CD62L negative effector/memory cells and (B) IFN gamma and TNF producing CD4 T cells.

Supplementary Figure 3

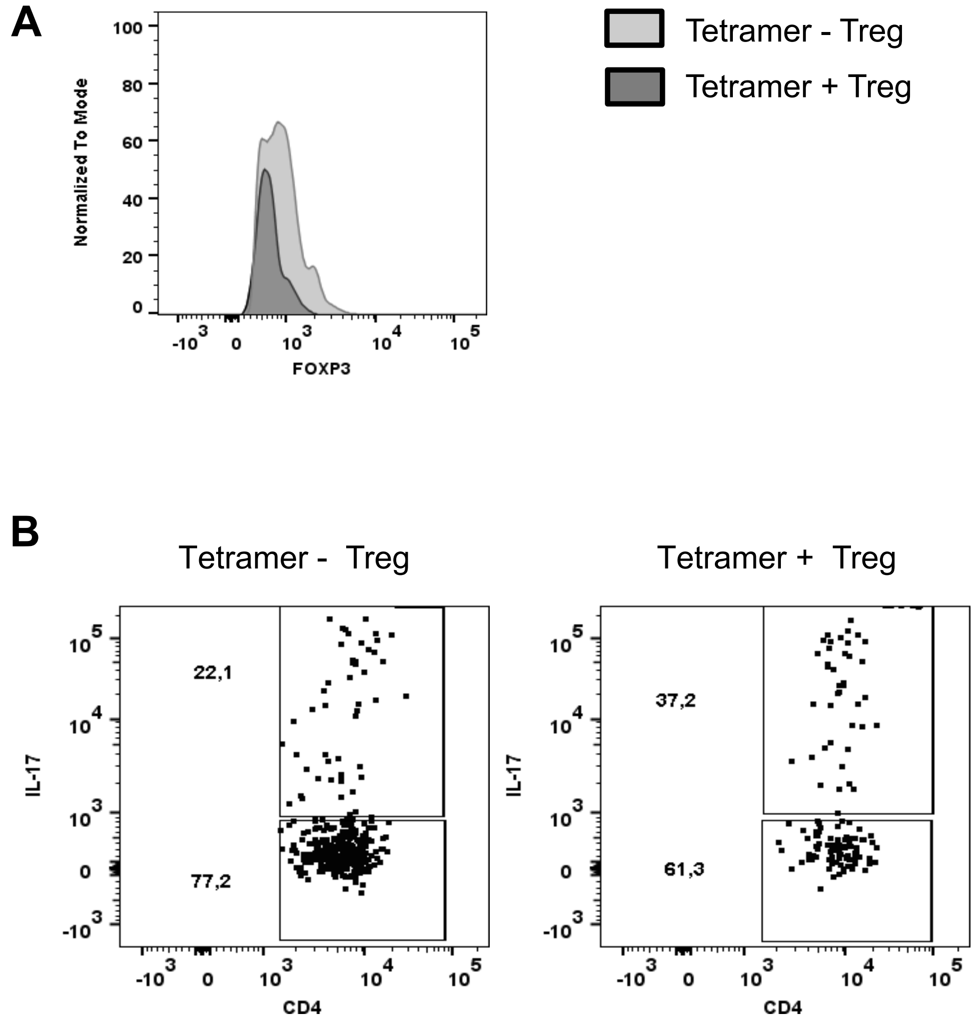

Characterization of hepatic T helper-like Tregs in Alb-iGP_Smarta mice.
(A) Representative histogram showing the Mean Fluorescence Intensity of Foxp3 expression in antigen-specific (Tetramer+) versus non-specific (Tetramer-) Tregs. (B) Representative dot plots of IL-17 expressing non-specific (left) versus antigen-specific (right) Tregs.
